# Supplementary material for: Evaluating the Therapeutic Efficacy of an Anti-BAFF Receptor Antibody Using a Rheumatoid Arthritis Mouse Model
Source: Antibodies (Basel). 2025 Oct 20;14(4):90. doi: 10.3390/antib14040090 (PMC12551023; doi:10.3390/antib14040090)
Supplement: Supplementary file 1 [file antibodies-14-00090-s001.zip › antibodies-3903532-supplementary.pdf]

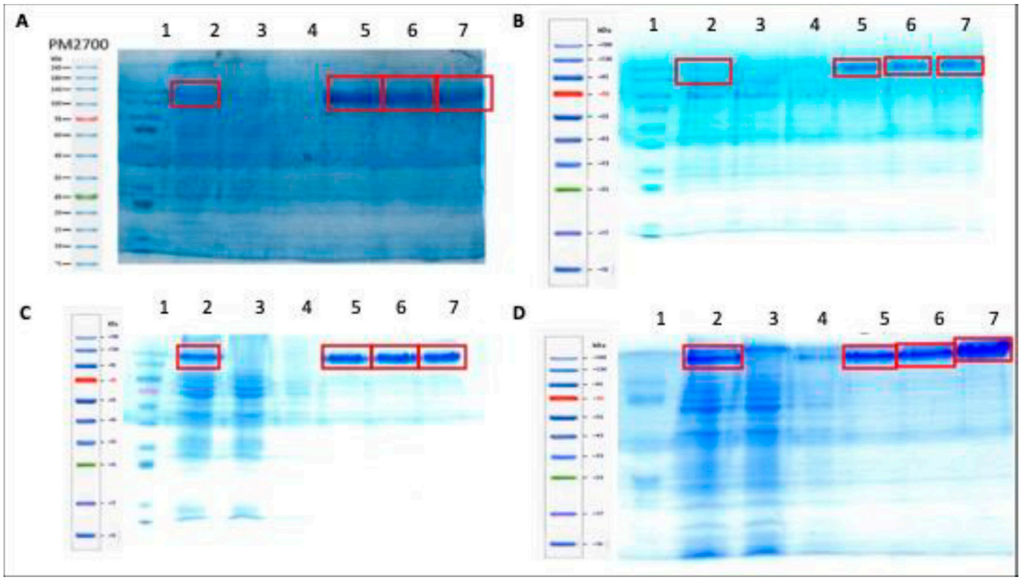

Supplemental Figure S1. SDS-PAGE analysis of anti BR3 antibodies expression and purification. V3-46s and V3-1 antibodies were cloned into pcDNA3.4 vectors to be expressed both as mouse IgG1 and mouse IgG2a in the mammalian Expi293F cell system. V3-46s and V3-1 mIgG1 were purified on a protein G affinity column and V3-46s and V3-1 mIgG2a were purified on a MabSelect© affinity column. **A. V3-46s mIgG2a, B. V3-46s mIgG1, C. V3-1 mIgG2a, D. V3-1 mIgG1.** In all the gels, 5 µg of protein samples were separated on a 12% SDS-polyacrylamide gel under non-reducing conditions. Gels were stained with InstaBlue protein stain reagent. Lane 1: protein marker. Lane 2: Un-purified conditioned medium (**CM**) collected on day 7 post transfection. Lane 3: MabSelect or protein G column flow-through (unbound fraction). Lane 4: column wash (PBS with Unbound proteins). Lane 5: Elution fractions 2-4. Lane 6: purified IgG after desalting on a PD10 column. Lane 7: purified IgG after centrifugation .

Control mice	RA mice+ PBS	RA mice+ IC	RA mice+ anti-BR3

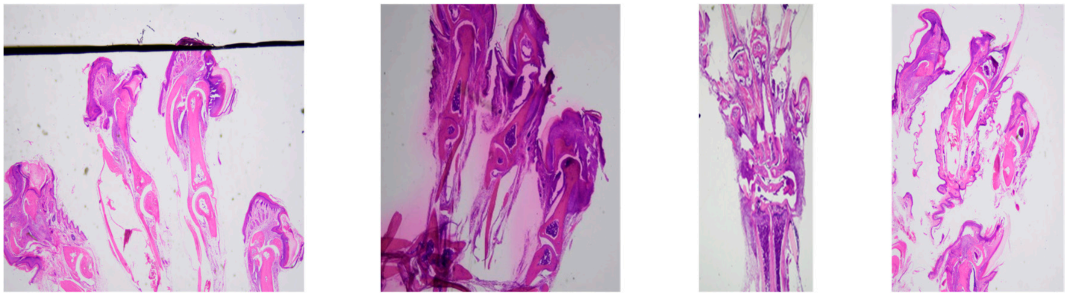

No inflammatory infiltrate, Pannus formation. Bone resorption or cartilage damage seen	Extensive and dense inflammatory infiltrate, Pannus formation. Bone resorption, and cartilage damage seen, involving all joints of the paw	Extensive and dense inflammatory infiltrate, Pannus formation. Bone resorption, and cartilage damage seen, involving all joints of the paw	A mild inflammatory infiltrate, a mild Pannus formation. No Bone resorption, and no cartilage damage seen, involving only one joint of the paw

Supplemental Figure S2. Histological evaluation of the joints. Following euthanization, the mice joints were dissected, and the samples were fixed in 4% paraformaldehyde for at least 24 hours before being decalcified. The joints were embedded in paraffin for sectioning, and 5-µm sections were then stained with H&E for histopathological examination. Representative histologic sections of whole paws from each experimental group are shown at ⊚10 magnification.
